# Supplementary material for: Computational design of bifaceted protein nanomaterials
Source: Nat Mater. 2025 Jul 31;24(10):1635–43. doi: 10.1038/s41563-025-02295-7 (PMC12407187; doi:10.1038/s41563-025-02295-7)
Supplement: Supplementary file 1 — Supplementary Tables 1 and 2. [file 41563_2025_2295_MOESM1_ESM.pdf]

nature materials

Supplementary information https://doi.org/10.1038/s41563-025-02295-7

Computational design of bifaceted protein nanomaterials

In the format provided by the authors and unedited

Table of Contents

Supplementary Table 1: Amino acid sequences of novel proteins used in this study 3
Supplementary Table 2: Cryo-EM data collection statistics for pD5-14 11

Supplementary Table 1. Amino acid sequences of novel proteins used in this study.

ID	Subunit	Sequence
pD5-14	A_6×His-tag	MGSLELALKALQILVNAAYVLAEIARDRGNEELLEKAARLAEEAARQAE<br>RIARQARKEGNLELALKALQILVNAAYVLAEIARDRGNEELLEYAARLAE<br>EAARQAIEIWAQAMEEGNQQLRTKAAHIILRAAEVLLEIARDRGNQELL<br>EKAASLVDAVAALQQAAAAILEGDVEKAVRAAQEAVKAAKEAGDNDML<br>RAVAIAALRIAKEAEKQGNVEVAVKAARVAVEAAKQAGDNDVLRKVAEQ<br>ALRIAKEAEKQGNVEVAVKAARVAVEAAKQAGDNDVLRKVADQALEIAK<br>AALEQGDIDVAQKAMDVAVEALTQAGGSGGSHHHHHH
	B	MGSPRLVLRALENMVRAAHTLAEIARDNGNEEWLERAARLAEEVA<br>RRAERLAREARKEGNLELALKALQILVNAAYVLAEIARDRGNEEELE<br>YAARLAEEAARQAIEIAAQAMEEGNLELALKALQIIVNAAYVLAEIAR<br>DRGNEELLEKAASLAEAAAALAEAIAAILEGDVEKAVRAAQEAVKAA<br>KEAGDNDMLRAVAIAALRIAKEAEKQGNVEVAVKAARVAVEAAKQA<br>GDNDVLRKVAEQALRIAKEAEKQGNVEVAVKAARVAVEAAKQAGD<br>NDVLRKVAEQALEIAKKAAEQGDVGVMQKAMDVALRAAGQAG
	mScarlet-I_C	MGSKGEAVIKEFMRFKVHMEGSMNGHEFEIEGEGEGRPYEGTQT<br>AKLKVTKGGPLPFSWDILSPQFMYGSRAFIKHPADIPDYYKQSFPE<br>GFKWERVMNFEDGGAVTVTQDTSLEDGTLIYKVKLRGTNFPPDGP<br>VMQKKTMGWEASTERLYPEDGVLKGDIKMALRLKDGGRYLADFKT<br>TYKAKKPVQMPGAYNVDRKLDITSHNEDYTVVEQYERSEGRHSTG<br>GMDELYKGSGSGPELFLQDLRSLVEAARILARLARQRGDEHALER<br>AARWAEQAARQAERLARQARKEGNLELALKALQILVNAAYVLAEIA<br>RDRGNEELLEYAARLAEEAARQAIEIAAQAMEEGNFELALEALEIIN<br>EAARVLARIAHHRGNQELLEKAASLTHASAALSRAIAAILEGDVEKA<br>VRAAQEAVKAAKEAGDNDMLRAVAIAALRIAKEAEKQGNVEVAVKA<br>ARVAVEAAKQAGDNDVLRLVSERALSIAASSVKQGNYEVKEKAIRV<br>AKEANKQAG
	mNeonGreen_D	MGSKGEEDNMASLPATHELHIFGSINGVDFDMVGQGTGNPNDGY<br>EELNLKSTKGDLQFSPWILVPHIGYGFHQYLPYPDGMSPFQAAMV<br>DGSGYQVHRTMQFEDGASLTVNYRYTYEGSHIKGEAQVKGTGFPA<br>DGPVMTNSLTAADWCRSKKTYPNDKTIISTFKWSYTTGNGKRYRS<br>TARTTYTFAKPMAANYLKNQPMYVFRKTELKHSKTELNFKEWQKA<br>FTDVMGMDELYKGSGSGPELFLQDLRSLVEAARILARLARQRGDE<br>HALERAARWAEQAARQAERLARQARKEGNLELALKALQILVNAAYV<br>LAEIARDRGNEELLEYAARLAEEAARQAIEIAAQAMEEGNFELALEA<br>LEIINEAARVLARIAHHRGNQELLEKAASLTHASAALSRAIAAILEGD<br>VEKAVRAAQEAVKAAKEAGDNDMLRAVAIAALRIAKEAEKQGNVEV<br>AVKAARVAVEAAKQAGDNDVLKRVSETLLSIAAEATKQGNSEVMEK<br>AIRVSEEAEKQAG
pD5$_{+25}$-52	mScarlet-I_C	MGSKGEAVIKEFMRFKVHMEGSMNGHEFEIEGEGEGRPYEGTQT<br>AKLKVTKGGPLPFSWDILSPQFMYGSRAFIKHPADIPDYYKQSFPE<br>GFKWERVMNFEDGGAVTVTQDTSLEDGTLIYKVKLRGTNFPPDGP<br>VMQKKTMGWEASTERLYPEDGVLKGDIKMALRLKDGGRYLADFKT<br>TYKAKKPVQMPGAYNVDRKLDITSHNEDYTVVEQYERSEGRHSTG<br>GMDELYKGSGSGPELFLQDLRSLVEAARILARLARQRGDEHALER<br>AARWAEQAARQAERLARQARKEGNLELALKALQILVNAAYVLAEIA<br>RDRGNEELLEYAARLAEEAARQAIEIAAQAMEEGNFELALEALEIIN<br>EAARVLARIAHHRGNQELLEKAASLTHASAALSRAIAAILEGDVEKA<br>VRAAQEAVKAAKEAGDNDMLRAVAIAALRIAKEAEKQGNVEVAVKA<br>ARVAVEAAKQAGDRELKAKGLWREARGHLELGNTDAFAESLAAML<br>EAVGEEERAALARELRELAVRLLEEGGSLADLRALLAALAALGDND

		VLRLVSERALSIAASSVKQGNYEVKEKAIRVAKEANKQAG
pD5$_{+25}$-41	mScarlet-I_C	MGSKGEAVIKEFMRFKVHMEGSMNGHEFEIEGEGEGRPYEGTQT<br>AKLKVTKGGPLPFSWDILSPQFMYGSRAFIKHPADIPDYYKQSFPE<br>GFKWERVMNFEDGGAVTVTQDTSLEDGTLIYKVKLRGTNFPPDGP<br>VMQKKTMGWEASTERLYPEDGVLKGDIKMALRLKDGGRYLADFKT<br>TYKAKKPVQMPGAYNVDRKLDITSHNEDYTVVEQYERSEGRHSTG<br>GMDELYKGSGSGPELFLQDLRSLVEAARILARLARQRGDEHALER<br>AARWAEQAARQAERLARQARKEGNLELALKALQILVNAAYVLAEIA<br>RDRGNEELLEYAARLAEEAARQAIEIAAQAMEEGNFELALEALEIIN<br>EAARVLARIAHHRGNQELLEKAASLTHASAALSRAIAAILEGDVEKA<br>VRAAQEAVKAAKEAGDNDMLRAVAIAALRIAKEAEKQGNVEVAVKA<br>ARVAVEAAKQAGDVDLREEGREGQARGLIKLGDREGARKVLKEISS<br>DAAEELLLELVTQRPLDVDLLRALMKHVEDNDVLRLVSERALSIAAS<br>SVKQGNYEVKEKAIRVAKEANKQAG
pD5$_{+50}$-98	mScarlet-I_C	MGSKGEAVIKEFMRFKVHMEGSMNGHEFEIEGEGEGRPYEGTQT<br>AKLKVTKGGPLPFSWDILSPQFMYGSRAFIKHPADIPDYYKQSFPE<br>GFKWERVMNFEDGGAVTVTQDTSLEDGTLIYKVKLRGTNFPPDGP<br>VMQKKTMGWEASTERLYPEDGVLKGDIKMALRLKDGGRYLADFKT<br>TYKAKKPVQMPGAYNVDRKLDITSHNEDYTVVEQYERSEGRHSTG<br>GMDELYKGSGSGPELFLQDLRSLVEAARILARLARQRGDEHALER<br>AARWAEQAARQAERLARQARKEGNLELALKALQILVNAAYVLAEIA<br>RDRGNEELLEYAARLAEEAARQAIEIAAQAMEEGNFELALEALEIIN<br>EAARVLARIAHHRGNQELLEKAASLTHASAALSRAIAAILEGDVEKA<br>VRAAQEAVKAAKEAGDNDMLRAVAIAALRIAKEAEKQGNVEVAVKA<br>ARVAVEAAKQAGNGALEAEAMKAELRAAARCMAEKGWSIEELEEL<br>LKEVEKLGTAARAECYKEAALELARAIIERPEDEEAVELLREVLDKIIE<br>LDASILNEILHELARAAIENEKHRAEMAAMAARVLRELEEVEGRDEL<br>RERLLELLLEESLEEEALRELARCWVLLARDDEEGFREALERLRSL<br>PLDAQVRRLRALAEAAEEQGDNDVLRLVSERALSIAASSVKQGNYE<br>VKEKAIRVAKEANKQAG
pD5$_{+50}$-61	mScarlet-I_C	MGSKGEAVIKEFMRFKVHMEGSMNGHEFEIEGEGEGRPYEGTQT<br>AKLKVTKGGPLPFSWDILSPQFMYGSRAFIKHPADIPDYYKQSFPE<br>GFKWERVMNFEDGGAVTVTQDTSLEDGTLIYKVKLRGTNFPPDGP<br>VMQKKTMGWEASTERLYPEDGVLKGDIKMALRLKDGGRYLADFKT<br>TYKAKKPVQMPGAYNVDRKLDITSHNEDYTVVEQYERSEGRHSTG<br>GMDELYKGSGSGPELFLQDLRSLVEAARILARLARQRGDEHALER<br>AARWAEQAARQAERLARQARKEGNLELALKALQILVNAAYVLAEIA<br>RDRGNEELLEYAARLAEEAARQAIEIAAQAMEEGNFELALEALEIIN<br>EAARVLARIAHHRGNQELLEKAASLTHASAALSRAIAAILEGDVEKA<br>VRAAQEAVKAAKEAGDNDMLRAVAIAALRIAKEAEKQGNVEVAVKA<br>ARVAVEAAKQAGDEELYQRALAMELASLLKRGDYEEAKELIEREPIT<br>EEATRIMCEILKNDLKALHRAAKLLLEAGLREGAKLFAKSMVEGVKK<br>GLGSKEGVKCLNELADELEFSQEECDLLADLLVEAGEEAYEEGDEE<br>ELEETVKLLAEWLEKCQISAATARRILEWMERLSLEQQVELLAAMV<br>KSQTDNDVLRLVSERALSIAASSVKQGNYEVKEKAIRVAKEANKQA<br>G
pD5$_{+75}$-134	mScarlet-I_C	MGSKGEAVIKEFMRFKVHMEGSMNGHEFEIEGEGEGRPYEGTQT<br>AKLKVTKGGPLPFSWDILSPQFMYGSRAFIKHPADIPDYYKQSFPE<br>GFKWERVMNFEDGGAVTVTQDTSLEDGTLIYKVKLRGTNFPPDGP<br>VMQKKTMGWEASTERLYPEDGVLKGDIKMALRLKDGGRYLADFKT<br>TYKAKKPVQMPGAYNVDRKLDITSHNEDYTVVEQYERSEGRHSTG<br>GMDELYKGSGSGPELFLQDLRSLVEAARILARLARQRGDEHALER<br>AARWAEQAARQAERLARQARKEGNLELALKALQILVNAAYVLAEIA<br>RDRGNEELLEYAARLAEEAARQAIEIAAQAMEEGNFELALEALEIIN

		EAARVLARIAHHRGNQELLEKAASLTHASAALSRAIAAILEGDVEKA<br>VRAAQEAVKAAKEAGDNDMLRAVAIAALRIAKEAEKQGNVEVAVKA<br>ARVAVEAAKQAGDDELTALGLAGQMKGLAKAGADLEELREVLDKLV<br>AKLEELEEVSEETKDRLTDVLEELLKREEYLELMREAMRRCKELGL<br>LEVLERLTERVIEEGASVEQLRELMEAYRELGLREEAQRLAVEAME<br>KLIEAGDLEGLLELLEEMLEAAEVFSRDLLIDLLLRAARVMLERMKE<br>ADLEERGEIAEQLVRTAEMLLEVVEDDAAALRELARLLEEAMRLLAE<br>LHTEDGIEWAGEQLAATAALAARALIALGDLEGFRRLMDELAEQLE<br>GDLELQVEVLQALRDAAADRDNDVLRLVSERALSIAASSVKQGNYE<br>VKEKAIRVAKEANKQAG
pD5$_{+75}$-251	mScarlet-I_C	MGSKGEAVIKEFMRFKVHMEGSMNGHEFEIEGEGEGRPYEGTQT<br>AKLKVTKGGPLPFSWDILSPQFMYGSRAFIKHPADIPDYYKQSFPE<br>GFKWERVMNFEDGGAVTVTQDTSLEDGTLIYKVKLRGTNFPPDGP<br>VMQKKTMGWEASTERLYPEDGVLKGDIKMALRLKDGGRYLADFKT<br>TYKAKKPVQMPGAYNVDRKLDITSHNEDYTVVEQYERSEGRHSTG<br>GMDELYKGSGSGPELFLQDLRSLVEAARILARLARQRGDEHALER<br>AARWAEQAARQAERLARQARKEGNLELALKALQILVNAAYVLAEIA<br>RDRGNEELLEYAARLAEEAARQAIEIAAQAMEEGNFELALEALEIIN<br>EAARVLARIAHHRGNQELLEKAASLTHASAALSRAIAAILEGDVEKA<br>VRAAQEAVKAAKEAGDNDMLRAVAIAALRIAKEAEKQGNVEVAVKA<br>ARVAVEAAKQAGDIELLCQAATGLCRGAAKLMVEGKELTDVEEFRE<br>LAAELKELKEELNRLEVARALASLYALLYMTGDEEAREELLEATREM<br>REMVASDRAKRQELLDELSEQVEALDGCGIVDEELLLLLAEEALEL<br>GDLELFEEVLKRALETDEETLERFIEILEEAGDLEALLLAAEVLLEDA<br>LERGREGAVRCLARLLRRLAELGAGADSLLALLQRCLRLPEDFVVR<br>ILEEVVEELGEETLLRLAQQLLEQGLEEALVALAGGMARAGHAEALL<br>ELLQQLEEQGLEELSIRVLQAIVETAKDNDVLRLVSERALSIAASSVK<br>QGNYEVKEKAIRVAKEANKQAG
pD5$_{+75}$-506	mScarlet-I_C	MGSKGEAVIKEFMRFKVHMEGSMNGHEFEIEGEGEGRPYEGTQT<br>AKLKVTKGGPLPFSWDILSPQFMYGSRAFIKHPADIPDYYKQSFPE<br>GFKWERVMNFEDGGAVTVTQDTSLEDGTLIYKVKLRGTNFPPDGP<br>VMQKKTMGWEASTERLYPEDGVLKGDIKMALRLKDGGRYLADFKT<br>TYKAKKPVQMPGAYNVDRKLDITSHNEDYTVVEQYERSEGRHSTG<br>GMDELYKGSGSGPELFLQDLRSLVEAARILARLARQRGDEHALER<br>AARWAEQAARQAERLARQARKEGNLELALKALQILVNAAYVLAEIA<br>RDRGNEELLEYAARLAEEAARQAIEIAAQAMEEGNFELALEALEIIN<br>EAARVLARIAHHRGNQELLEKAASLTHASAALSRAIAAILEGDVEKA<br>VRAAQEAVKAAKEAGDNDMLRAVAIAALRIAKEAEKQGNVEVAVKA<br>ARVAVEAAKQAGDEELYIEALAGEAKELVKLGDIEGAAKVFLEMVER<br>DLEKAAETAKEIIEEYQEEATELFLLLGKENLEALVEILKALKKMESM<br>TFEELLKAAKILGKVILEDESVSEEVCKIFIEFVKKILIQSEKATLEELL<br>EMAEVLQKILEKRELSEDIRREFVLCLSQVLLQIAKRIKELDVEEAEE<br>KIEELIEKLEKLQSEEELDEELMGQTCLILLETCITVLPIDDKRIEKISR<br>LVELLVSLSNIDIHRKACELLDAEIDKLVDAPLEYKLTMLACLRDVAAA<br>AGDNDVLRLVSERALSIAASSVKQGNYEVKEKAIRVAKEANKQAG
pD5$_{+100}$-9add	mScarlet-I_C	MGSKGEAVIKEFMRFKVHMEGSMNGHEFEIEGEGEGRPYEGTQT<br>AKLKVTKGGPLPFSWDILSPQFMYGSRAFIKHPADIPDYYKQSFPE<br>GFKWERVMNFEDGGAVTVTQDTSLEDGTLIYKVKLRGTNFPPDGP<br>VMQKKTMGWEASTERLYPEDGVLKGDIKMALRLKDGGRYLADFKT<br>TYKAKKPVQMPGAYNVDRKLDITSHNEDYTVVEQYERSEGRHSTG<br>GMDELYKGSGSGPELFLQDLRSLVEAARILARLARQRGDEHALER<br>AARWAEQAARQAERLARQARKEGNLELALKALQILVNAAYVLAEIA<br>RDRGNEELLEYAARLAEEAARQAIEIAAQAMEEGNFELALEALEIIN<br>EAARVLARIAHHRGNQELLEKAASLTHASAALSRAIAAILEGDVEKA<br>VRAAQEAVKAAKEAGDNDMLRAVAIAALRIAKEAEKQGNVEVAVKA

		ARVAVEAAKQAGDERLQSEALLAEAKALREMGDFDGFAETLRELLE<br>LPSAREVIKLEELLEFAQYLTQQRIAGADPELCDELLQELLEKFEEH<br>RLEMLTVIALELAKNPAFAERLKEVFRELVELGISEEKKKELLIYMFR<br>QGLVEEVLELCLEENVSEEILRELGLLAIDDPEVFEKLIDVMRTLGYT<br>RLARELLAQRLESLLVRPPLSPQQKQEMLDTAIRLMRECRELGEMS<br>PRERQLLFQCGQVFIHDLEAFQQLCTELQKLREAGIPEVNTLAFQLL<br>QYLLQNKDQLDVEDFAQQVEQLLLILDLEQFKEAVEVILQSLKSAPL<br>ELQVEVLEAIRRAAEKKGDNDVLRLVSERALSIAASSVKQGNYEVK<br>EKAIRVAKEANKQAG
pD5$_{+100}$-46	mScarlet-I_C	MGSKGEAVIKEFMRFKVHMEGSMNGHEFEIEGEGEGRPYEGTQT<br>AKLKVTKGGPLPFSWDILSPQFMYGSRAFIKHPADIPDYYKQSFPE<br>GFKWERVMNFEDGGAVTVTQDTSLEDGTLIYKVKLRGTNFPPDGP<br>VMQKKTMGWEASTERLYPEDGVLKGDIKMALRLKDGGRYLADFKT<br>TYKAKKPVQMPGAYNVDRKLDITSHNEDYTVVEQYERSEGRHSTG<br>GMDELYKGSGSGPELFLQDLRSLVEAARILARLARQRGDEHALER<br>AARWAEQAARQAERLARQARKEGNLELALKALQILVNAAYVLAEIA<br>RDRGNEELLEYAARLAEEAARQAIEIAAQAMEEGNFELALEALEIIN<br>EAARVLARIAHHRGNQELLEKAASLTHASAALSRAIAAILEGDVEKA<br>VRAAQEAVKAAKEAGDNDMLRAVAIAALRIAKEAEKQGNVEVAVKA<br>ARVAVEAAKQAGDERLQAEALLAKAEALMEMGDFDGFAATLDELL<br>RLPEARAVVELERLKRFARHLTQQMIAGADPERCERLLQQLLEKFE<br>EHRLEMLTVIALELAKNPEFAEKLKEVFRELVELGISEEEKKELLLYM<br>FEQGLVELVLELCREENVSEEILEELAEKAIDNPEVFFKLIEVMRELG<br>YTELARKKLAERLKKLLEKPPLSPEEKKEMLDTAIELMEEARELGKL<br>TEEERELLYECMLVFVFDEEAFKKLCEELKKLREAGIEEVNELAYES<br>LLELLKRRDELDVDDFAKQVRDLLLILDEERFKEAVEEILKSLESAPL<br>ELQVKVLEALRKAAKEKGDNDVLRLVSERALSIAASSVKQGNYEVK<br>EKAIRVAKEANKQAG
pD5$_{+100}$-48	mScarlet-I_C	MGSKGEAVIKEFMRFKVHMEGSMNGHEFEIEGEGEGRPYEGTQT<br>AKLKVTKGGPLPFSWDILSPQFMYGSRAFIKHPADIPDYYKQSFPE<br>GFKWERVMNFEDGGAVTVTQDTSLEDGTLIYKVKLRGTNFPPDGP<br>VMQKKTMGWEASTERLYPEDGVLKGDIKMALRLKDGGRYLADFKT<br>TYKAKKPVQMPGAYNVDRKLDITSHNEDYTVVEQYERSEGRHSTG<br>GMDELYKGSGSGPELFLQDLRSLVEAARILARLARQRGDEHALER<br>AARWAEQAARQAERLARQARKEGNLELALKALQILVNAAYVLAEIA<br>RDRGNEELLEYAARLAEEAARQAIEIAAQAMEEGNFELALEALEIIN<br>EAARVLARIAHHRGNQELLEKAASLTHASAALSRAIAAILEGDVEKA<br>VRAAQEAVKAAKEAGDNDMLRAVAIAALRIAKEAEKQGNVEVAVKA<br>ARVAVEAAKQAGDERLQAEALLGKAEALRKMGDFDGFAETLEELLT<br>LPSAVEVMKLEELLEFARYLTEQKIAGADPERCDRLLEELLEKFEEH<br>RLEMLKVIALELAKNPAFAEELKEVFRELVRLGMSEEEKKELLIYMF<br>EQGLVELVLELCLEEKVSEEILRELGEKAIDNPEVFFKLIDVMKELGY<br>IELARDMLAKKLESLLVKPPLSPEEKEELLKTAIELMDCAKELGYLTE<br>REKELLFECGEVFVFDQEAMEKLCEKLQELREAGIEEVNELAFELL<br>EYLLEHADELDVDDFAKQVEALLRILDKEQFKEAVEVIWESLESAPL<br>ERQVKILEAIRRAAVEAGDNDVLRLVSERALSIAASSVKQGNYEVKE<br>KAIRVAKEANKQAG
pD5$_{+100}$-71	mScarlet-I_C	MGSKGEAVIKEFMRFKVHMEGSMNGHEFEIEGEGEGRPYEGTQT<br>AKLKVTKGGPLPFSWDILSPQFMYGSRAFIKHPADIPDYYKQSFPE<br>GFKWERVMNFEDGGAVTVTQDTSLEDGTLIYKVKLRGTNFPPDGP<br>VMQKKTMGWEASTERLYPEDGVLKGDIKMALRLKDGGRYLADFKT<br>TYKAKKPVQMPGAYNVDRKLDITSHNEDYTVVEQYERSEGRHSTG<br>GMDELYKGSGSGPELFLQDLRSLVEAARILARLARQRGDEHALER<br>AARWAEQAARQAERLARQARKEGNLELALKALQILVNAAYVLAEIA<br>RDRGNEELLEYAARLAEEAARQAIEIAAQAMEEGNFELALEALEIIN

		EAARVLARIAHHRGNQELLEKAASLTHASAALSRAIAAILEGDVEKA<br>VRAAQEAVKAAKEAGDNDMLRAVAIAALRIAKEAEKQGNVEVAVKA<br>ARVAVEAAKQAGDEELEAEALLGKAEALMEMGDFEGFAETLRQLL<br>ELPSARRVMKLERLKRFARHLTQQMIAGADPELCEELLRQLLEKFR<br>EHRLECLEVIALELAKNPEFAEKWKEVFRELVELGISEEKRKELLIYA<br>FEQGLVEEVLELCREERVSEEILRELGLLAIDDPEVFFRLIEVMRELG<br>YLELAREMLAEKLRSLLEKPPLSPEEKEELLKTAIRLMECCRELGYM<br>TPEERELLFECGQVFVFDEEAFKKLCEKLKELREAGIEEVNELAFEL<br>LQYLLEHRDELDVEELAEQIERLCLILNEEQFKEAVELILESLKSAPL<br>ELQVEVLEALRNAAKEKGDNDVLRLVSERALSIAASSVKQGNYEVK<br>EKAIRVAKEANKQAG
pD5$_{+50/25°}$-344	mScarlet-I_C	MGSKGEAVIKEFMRFKVHMEGSMNGHEFEIEGEGEGRPYEGTQT<br>AKLKVTKGGPLPFSWDILSPQFMYGSRAFIKHPADIPDYYKQSFPE<br>GFKWERVMNFEDGGAVTVTQDTSLEDGTLIYKVKLRGTNFPPDGP<br>VMQKKTMGWEASTERLYPEDGVLKGDIKMALRLKDGGRYLADFKT<br>TYKAKKPVQMPGAYNVDRKLDITSHNEDYTVVEQYERSEGRHSTG<br>GMDELYKGSGSGPELFLQDLRSLVEAARILARLARQRGDEHALER<br>AARWAEQAARQAERLARQARKEGNLELALKALQILVNAAYVLAEIA<br>RDRGNEELLEYAARLAEEAARQAIEIAAQAMEEGNFELALEALEIIN<br>EAARVLARIAHHRGNQELLEKAASLTHASAALSRAIAAILEGDVEKA<br>VRAAQEAVKAAKEAGDNDMLRAVAIAALRIAKEAEKQGNVEVAVKA<br>ARVAVEAAKQAGDVALLAKGLVAKARSLVELGADLEEIKEVIREAIRA<br>MVECKDEDCMRELAELMLELQKKGEKELFEFALREICRQLKDSDTE<br>FRVRFLLICREVGLDVETLGRLTEELIRQGNNASELLSLLLERALER<br>GDEDLADLVEEIAIKHRETAVAVARRLARLAEERDAATSARCRERLD<br>RLAAHFADDEELGKALVEEAARRLRRLERIDEARQYYLETLERAAR<br>LGLKTIEELAALGTEVLMEKEEMKKEDVEKLLDALEEAEELKDSALV<br>AALEALRKKAVEKGDNDVLRLVSERALSIAASSVKQGNYEVKEKAIR<br>VAKEANKQAG
pD5$_{+50/25°}$-218	mScarlet-I_C	MGSKGEAVIKEFMRFKVHMEGSMNGHEFEIEGEGEGRPYEGTQT<br>AKLKVTKGGPLPFSWDILSPQFMYGSRAFIKHPADIPDYYKQSFPE<br>GFKWERVMNFEDGGAVTVTQDTSLEDGTLIYKVKLRGTNFPPDGP<br>VMQKKTMGWEASTERLYPEDGVLKGDIKMALRLKDGGRYLADFKT<br>TYKAKKPVQMPGAYNVDRKLDITSHNEDYTVVEQYERSEGRHSTG<br>GMDELYKGSGSGPELFLQDLRSLVEAARILARLARQRGDEHALER<br>AARWAEQAARQAERLARQARKEGNLELALKALQILVNAAYVLAEIA<br>RDRGNEELLEYAARLAEEAARQAIEIAAQAMEEGNFELALEALEIIN<br>EAARVLARIAHHRGNQELLEKAASLTHASAALSRAIAAILEGDVEKA<br>VRAAQEAVKAAKEAGDNDMLRAVAIAALRIAKEAEKQGNVEVAVKA<br>ARVAVEAAKQAGDVGLLALGLIAEARSLIELGAELEEIKEVIEEAIEAM<br>VEDGRKESMKALAELAKYLQEKGEKELFEYLLEKLAEELKDKDTEF<br>KVEVLLIFSEVGLDIETIEKLTRKLIEEGNNAAELLSELLARALERGDQ<br>ELADLVREIAIEKRETVTAVAAELAARAQREDAATAARCEALLDELLA<br>HFAEDEELGRTWVEQAAARLEELERIDDAIQFMLDTLERAAELGLR<br>TIGDLAAKLMEVLMEKEEMKRADIEKLLDRLLEAESLRAEALVALLE<br>AARDKAIEKGDNDVLRLVSERALSIAASSVKQGNYEVKEKAIRVAKE<br>ANKQAG
pD5-14_rd106	A	MGSSELLRKAALLAAEAAEQAARIAKQAAKGELKNLELALKALQILV<br>NAAYVLAEIARDRGEKPEIEEILPELRKLAKEAEEEAKEAKKEIEKAT<br>EQGLELALKALQILVNAAYVLAEIARDRGNEELLEIAAKLAEEAAELAI<br>EVWALAMERGNQQLRTKAAHIILRAAEVLLEIARDRGNQELLEKAA<br>SLVDAVAALQQAAAAILEGDVEKAVRAAQEAVKAAKEAGDNDMLRA<br>VAIAALRIAKEAEKQGNVEVAVKAARVAVEAAKQAGDNDVLRKVAE<br>QALRIAKEAEKQGNVEVAVKAARVAVEAAKQAGDNDVLRKVADQAL<br>EIAKAALEQGDIDVAQKAMDVAVEALTQAGGSGGSHHHHHH

	B	MGSEEWLTRAALLALEVAVRAARLAAEAAKGVRENPRLVLRALEN MVRAAHTLAEIARDNGPGTPEREEEIEPLIEELEKELERAKKAFEEY GKNPEGLELALKALQILVNAAYVLAEIARDRGNERLLEAAAKLAESA AELAIKVAAEAMELGNLELALKALQIIVNAAYVLAEIARDRGNEELLE KAASLAEAAAALAEAIAAILEGDVEKAVRAAQEAVKAAKEAGDNDM LRAVAIAALRIAKEAEKQGNVEVAVKAARVAVEAAKQAGDNDVLRKV AEQALRIAKEAEKQGNVEVAVKAARVAVEAAKQAGDNDVLRKVAE QALEIAKKAAEQGDVGVMQKAMDVALRAAGQAG
	mScarlet-I_C	MGSKGEAVIKEFMRFKVHMEGSMNGHEFEIEGEGEGRPYEGTQT AKLKVTKGGPLPFSWDILSPQFMYGSRAFIKHPADIPDYYKQSFPE GFKWERVMNFEDGGAVTVTQDTSLEDGTLIYKVKLRGTNFPPDGP VMQKKTMGWEASTERLYPEDGVLKGDIKMALRLKDGGRYLADFKT TYKAKKPVQMPGAYNVDRKLDITSHNEDYTVVEQYERSEGRHSTG GMDELYKGSGSGTHALVRAAKWAAQAAEQALRLAVQAAKGTVEN PELFLQDLRSLVEAARILARLARQRGEDTPEGDEELDRLAERLKEQ LRRLAEEFAAAAANSENLELALKALQILVNAAYVLAEIARDRGWERV LDAAAELAEAAARMAIEIAARAMEEGNFELALEALEIINEAARVLARI AHHRGNQELLEKAASLTHASAALSRAIAAILEGDVEKAVRAAQEAVK AAKEAGDNDMLRAVAIAALRIAKEAEKQGNVEVAVKAARVAVEAAK QAGDNDVLRLVSERALSIAASSVKQGNYEVKEKAIRVAKEANKQAG
	mNeonGreen_D	MGSKGEEDNMASLPATHELHIFGSINGVDFDMVGQGTGNPNDGY EELNLKSTKGDLQFSPWILVPHIGYGFHQYLPYPDGMSPFQAAMV DGSGYQVHRTMQFEDGASLTVNYRYTYEGSHIKGEAQVKGTGFPA DGPVMTNSLTAADWCRSKKTYPNDKTIISTFKWSYTTGNGKRYRS TARTTYTFAKPMAANYLKNQPMYVFRKTELKHSKTELNFKEWQKA FTDVMGMDELYKGSGSGTHALVRAAKWAAQAAEQALRLAVQAAK GTVENPELFLQDLRSLVEAARILARLARQRGEDTPEGDEELDRLAE RLKEQLRRLAEEFAAAAANSENLELALKALQILVNAAYVLAEIARDR GWERVLDAAAELAEAAARMAIEIAARAMEEGNFELALEALEIINEAA RVLARIAHHRGNQELLEKAASLTHASAALSRAIAAILEGDVEKAVRA AQEAVKAAKEAGDNDMLRAVAIAALRIAKEAEKQGNVEVAVKAARV AVEAAKQAGDNDVLKRVSETLLSIAAEATKQGNSEVMEKAIRVSEE AEKQAG
pD5-14_rd47	A	MGSGELLFKAAKLALEAAKQAARIAAQAIKGEDNNLELALKALQILV NAAYVLAEIARDRGEKELTEEQKRELKELIEELKKELREALEVAKTEL GENLELALKALQILVNAAYVLAEIARDRGDEELLEAAAEIAESAAEIAI LVWAKAMEQGNQQLRTKAAHIILRAAEVLLEIARDRGNQELLEKAA SLVDAVAALQQAAAAILEGDVEKAVRAAQEAVKAAKEAGDNDMLRA VAIAALRIAKEAEKQGNVEVAVKAARVAVEAAKQAGDNDVLRKVAE QALRIAKEAEKQGNVEVAVKAARVAVEAAKQAGDNDVLRKVADQAL EIAKAALEQGDIDVAQKAMDVAVEALTQAGGSGGSHHHHHH
	B	MGSAEWLERAAELALEVAERAARLAAEAWRTGVEEPRLVLRALEN MVRAAHTLAEIARDNGERPDLEEIRERLEELAERLREELERAKRVA KEEEGKNLELALKALQILVNAAYVLAEIARDRGIELLLEAAAELAETA AELAIIAAAKAMEQGNLELALKALQIIVNAAYVLAEIARDRGNEELLE KAASLAEAAAALAEAIAAILEGDVEKAVRAAQEAVKAAKEAGDNDM LRAVAIAALRIAKEAEKQGNVEVAVKAARVAVEAAKQAGDNDVLRKV AEQALRIAKEAEKQGNVEVAVKAARVAVEAAKQAGDNDVLRKVAE QALEIAKKAAEQGDVGVMQKAMDVALRAAGQAG
	mScarlet-I_C	MGSKGEAVIKEFMRFKVHMEGSMNGHEFEIEGEGEGRPYEGTQT AKLKVTKGGPLPFSWDILSPQFMYGSRAFIKHPADIPDYYKQSFPE GFKWERVMNFEDGGAVTVTQDTSLEDGTLIYKVKLRGTNFPPDGP VMQKKTMGWEASTERLYPEDGVLKGDIKMALRLKDGGRYLADFKT

		TYKAKKPVQMPGAYNVDRKLDITSHNEDYTVVEQYERSEGRHSTG GMDELYKGSGSGDHALDRAALWALQAAIQAARLAAQAVLGTLQQP ELFLQDLRSLVEAARILARLARQRGDLGGEEAREELREWLRELREA LREAERVVAEHLSPVNLELALKALQILVNAAYVLAEIARDRGDEELLE AAAELAERAAEMAIRIAALAMEEGNFELALEALEIINEAARVLARIAH HRGNQELLEKAASLTHASAALSRAIAAILEGDVEKAVRAAQEAVKAA KEAGDNDMLRAVAIAALRIAKEAEKQGNVEVAVKAARVAVEAAKQA GDNDVLRLVSERALSIAASSVKQGNYEVKEKAIRVAKEANKQAG
	mNeonGreen_D	MGSKGEEDNMASLPATHELHIFGSINGVDFDMVGQGTGNPNDGY EELNLKSTKGDLQFSPWILVPHIGYGFHQYLPYPDGMSPFQAAMV DGSGYQVHRTMQFEDGASLTVNYRYTYEGSHIKGEAQVKGTGFPA DGPVMTNSLTAADWCRSKKTYPNDKTIISTFKWSYTTGNGKRYRS TARTTYTFAKPMAANYLKNQPMYVFRKTELKHSKTELNFKEWQKA FTDVMGMDELYKGSGSGDHALDRAALWALQAAIQAARLAAQAVLG TLQQPELFLQDLRSLVEAARILARLARQRGDLGGEEAREELREWLR ELREALREAERVVAEHLSPVNLELALKALQILVNAAYVLAEIARDRG DEELLEAAAELAERAAEMAIRIAALAMEEGNFELALEALEIINEAARV LARIAHHRGNQELLEKAASLTHASAALSRAIAAILEGDVEKAVRAAQ EAVKAAKEAGDNDMLRAVAIAALRIAKEAEKQGNVEVAVKAARVAV EAAKQAGDNDVLKRVSETLLSIAAEATKQGNSEVMEKAIRVSEEAE KQAG
pD5 (in Figure 5; derived from pD5-14_rd47)	C (without mScarlet)	MGSDHALDRAALWALQAAIQAARLAAQAVLGTLQQPELFLQDLRSL VEAARILARLARQRGDLGGEEAREELREWLRELREALREAERVVA EHLSPVNLELALKALQILVNAAYVLAEIARDRGDEELLEAAAELAERA AEMAIRIAALAMEEGNFELALEALEIINEAARVLARIAHHRGNQELLE KAASLTHASAALSRAIAAILEGDVEKAVRAAQEAVKAAKEAGDNDM LRAVAIAALRIAKEAEKQGNVEVAVKAARVAVEAAKQAGDNDVLRLV SERALSIAASSVKQGNYEVKEKAIRVAKEANKQAG
	D (without mNeonGreen)	MGSDHALDRAALWALQAAIQAARLAAQAVLGTLQQPELFLQDLRSL VEAARILARLARQRGDLGGEEAREELREWLRELREALREAERVVA EHLSPVNLELALKALQILVNAAYVLAEIARDRGDEELLEAAAELAERA AEMAIRIAALAMEEGNFELALEALEIINEAARVLARIAHHRGNQELLE KAASLTHASAALSRAIAAILEGDVEKAVRAAQEAVKAAKEAGDNDM LRAVAIAALRIAKEAEKQGNVEVAVKAARVAVEAAKQAGDNDVLKRV SETLLSIAAEATKQGNSEVMEKAIRVSEEAEKQAG
	Neo-2/15_B	MPKKKIQLHAEHALYDALMILNIVKTNSPPAEEKLEDYAFNFELILEEI ARLFESGDQKDEAEKAKRMKEWMKRIKTTASEDEQEEMANAIITIL QSWIFSGGSGSGGGSGSAEWLERAAELALEVAERAARLAAEAWR TGVEEPRLVLRALENMVRAAHTLAEIARDNGERPDLEEIRERLEELA ERLREELERAKRVAKEEEGKNLELALKALQILVNAAYVLAEIARDRGI ELLLEAAAELAETAAELAIIAAAKAMEQGNLELALKALQIIVNAAYVLA EIARDRGNEELLEKAASLAEAAAALAEAIAAILEGDVEKAVRAAQEA VKAAKEAGDNDMLRAVAIAALRIAKEAEKQGNVEVAVKAARVAVEA AKQAGDNDVLRKVAEQALRIAKEAEKQGNVEVAVKAARVAVEAAK QAGDNDVLRKVAEQALEIAKKAAEQGDVGVMQKAMDVALRAAGQ AG
	4-1BB_mb1_B	MSGKATLEDLIALYEKGAAILEQIKPLVEKDMGLSNRTVATAIEEIKEA IKRVKKSGRIVYPIGLSIADNILALAQYYGNEKVAALAKELQKVGDAA AAAVAEMVAAEEAGGSGSGGGSGSAEWLERAAELALEVAERAARL AAEAWRTGVEEPRLVLRALENMVRAAHTLAEIARDNGERPDLEEIR ERLEELAERLREELERAKRVAKEEEGKNLELALKALQILVNAAYVLA EIARDRGIELLLEAAAELAETAAELAIIAAAKAMEQGNLELALKALQII VNAAYVLAEIARDRGNEELLEKAASLAEAAAALAEAIAAILEGDVEKA

		VRAAQEAVKAAKEAGDNDMLRAVAIAALRIAKEAEKQGNVEVAVKAARVAVEAAKQAGDNDVLRKVAEQALRIAKEAEKQGNVEVAVKAARVAVEAAKQAGDNDVLRKVAEQALEIAKKAAEQGDVGVMQKAMDVALRAAGQAG

Supplementary Table 2. Cryo-EM data collection statistics for pD5-14.

**Data Collection**	
Microscope	Titan Krios (FEI)
Voltage (kV)	300
Detector	K3 (Gatan)
Energy Filter	BioQuantum Gif (Gatan)
Recording mode	Counting
Magnification	105,000×
Movie micrograph pixel size (Å)	0.843
Dose rate (e^{-}/Å^{2}/s)	11.31
No. of frames per movie micrograph	79
Frame exposure time (ms)	0.0505
Movie micrograph exposure time (s)	3.997
Total dose (e^{-}/Å^{2})	45.21
Under focus range (µm)	0.8–1.8
Total number of movies collected	4871
Total number of movies used	4854
**Map Processing**	
Extraction Box Size (pix)	800
Fourier crop to Box Size (pix)	400
Initial particle images (no.)	640,897
Final particle images (no.)	209,004
Map resolution (Å)	4.30
FCS threshold	0.143
Map resolution range (Å)	3.71–5.28
**Refinement**	
Initial model used	Design Model
Map resolution (Å)	4.30
FCS threshold	0.143
Model resolution range (Å)	3.71–5.28
Map sharpening B factor	239.90
Model composition	
Non-hydrogen atoms	44,930
Protein Residues	9,060
Ligands	N/A
B factors (Å)	
Protein	DeepEMhancer
Ligands	N/A
R.M.S. deviations	
Bond lengths (Å) (# > 4 σ)	0.007 (0)
Bond angles (°) (# > 4 σ)	1.471 (1)
Validation	
MolProbity score	0.50
Clashscore	0.00
Rotamer Outliers (%)	0.00
Ramachandran plot	
Favored (%)	99.39
Allowed (%)	0.61
Outliers (%)	0.00
