# Supplementary figures and images for: Computational design of bifaceted protein nanomaterials
Source: Nat Mater. 2025 Jul 31;24(10):1635–43. doi: 10.1038/s41563-025-02295-7 (PMC12407187; doi:10.1038/s41563-025-02295-7)

**b**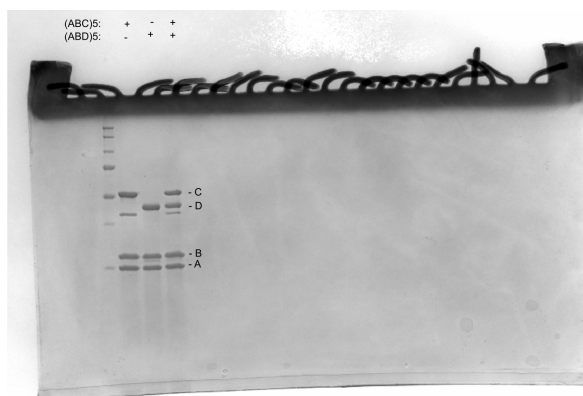**c** $(ABC)_5$ 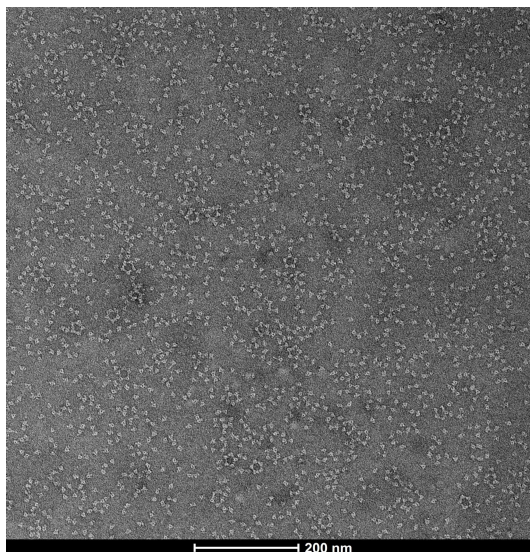**d** $(ABD)_5$ 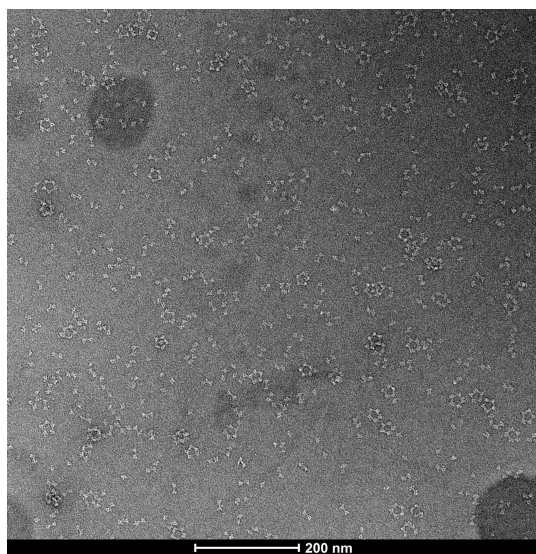**e** $(ABC)_5 + (ABD)_5$ 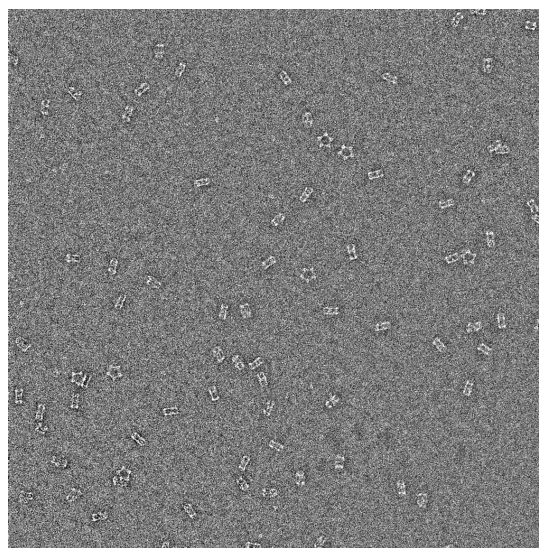

Supplement: Supplementary file 2 — Unprocessed gel and nsEM images [file 41563_2025_2295_MOESM2_ESM.pdf]

$(ABC)_5$

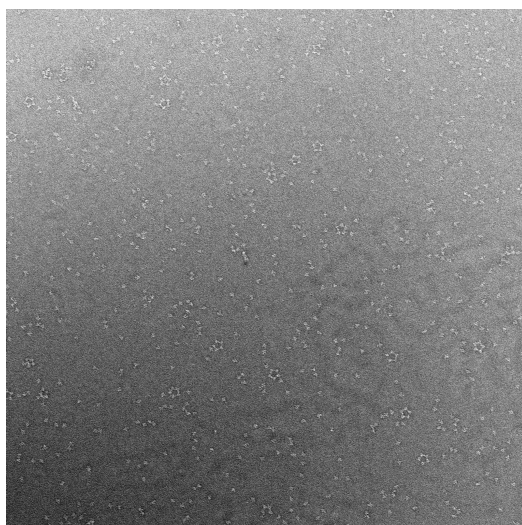

pD5<sub>+25</sub>-52

$(ABC)_5 + (ABD)_5$

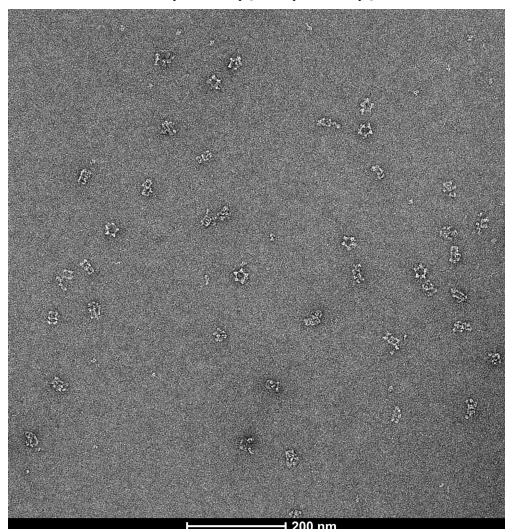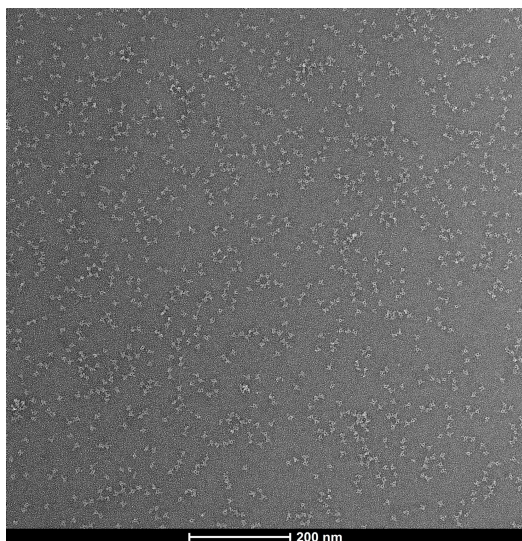

pD5<sub>+50</sub>-98

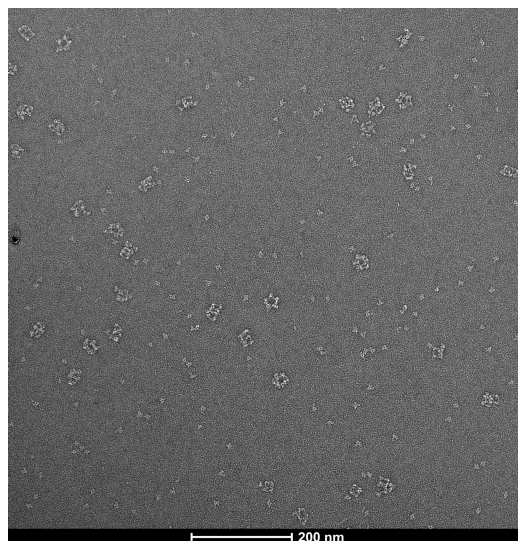

pD5<sub>+75</sub>-134

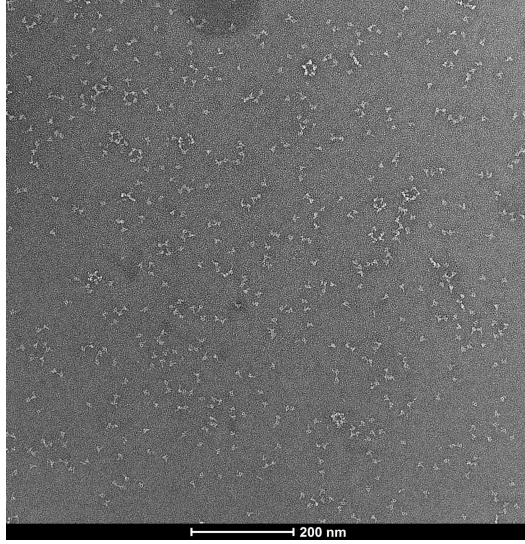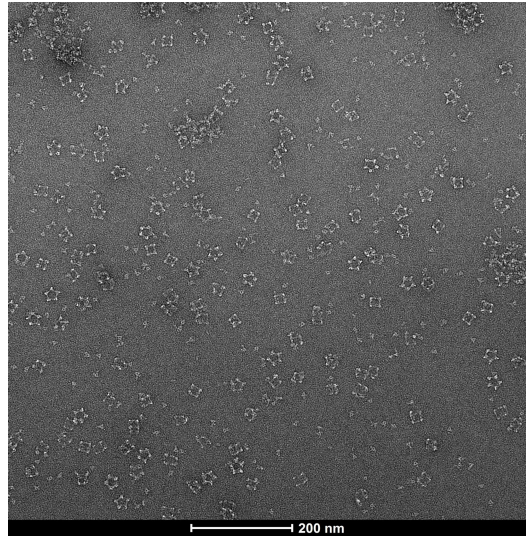

pD5<sub>+100</sub>-9add

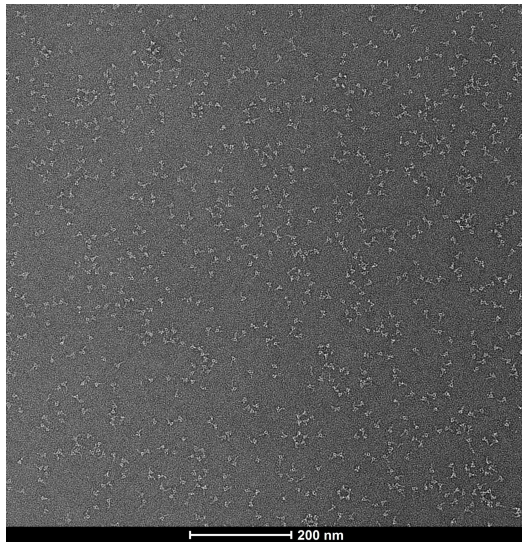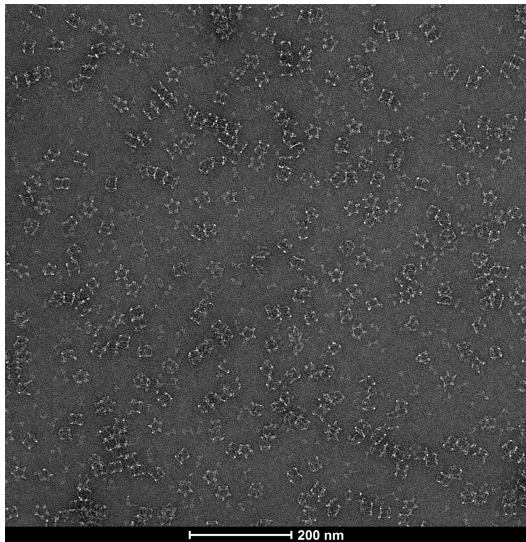

pD5<sub>+50/25</sub>-344

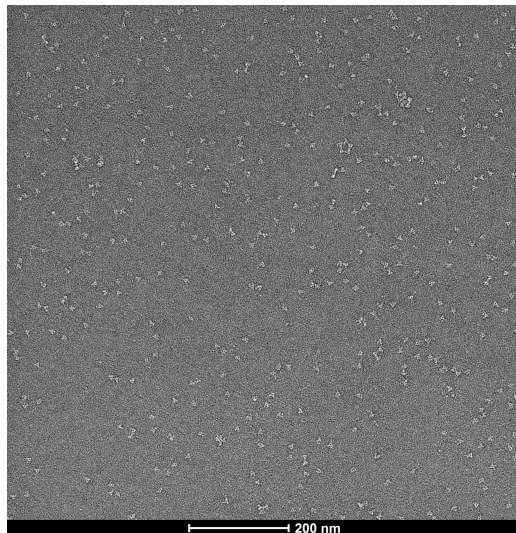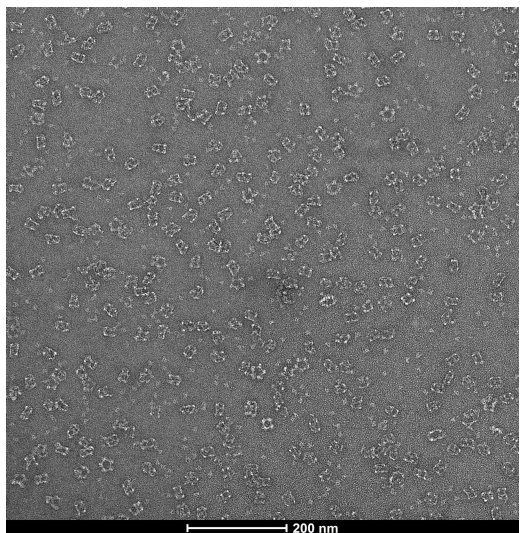

Supplement: Supplementary file 4 — Unprocessed nsEM images. [file 41563_2025_2295_MOESM4_ESM.pdf]

Neo-2/15-pD5-41bb\_mb1

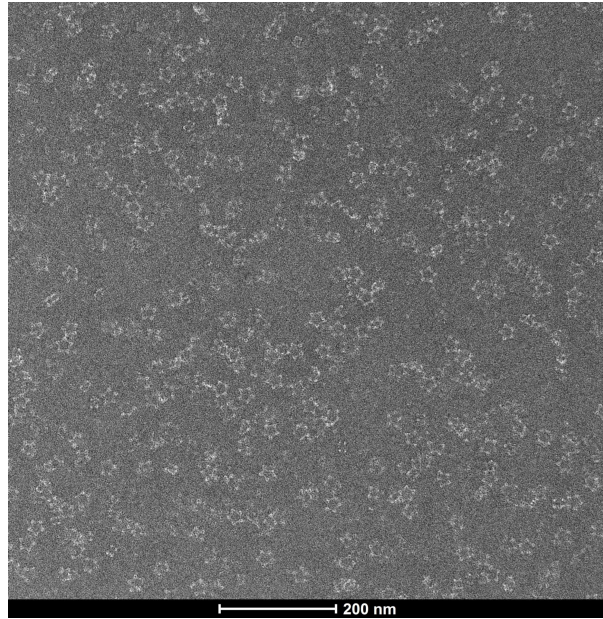

Supplement: Supplementary file 5 — Unprocessed nsEM image. [file 41563_2025_2295_MOESM5_ESM.pdf]
